# CT-guided injection in coccydynia: outcome prediction using MRI findings and CT-injection characteristics

**DOI:** 10.1007/s00330-026-12615-3

**Published:** 2026-05-13

**Authors:** Georg Wilhelm Kajdi, Thomas Marth, Christoph Germann, Mazda Farshad, Reto Sutter

**Affiliations:** 1 Department of Radiology, Balgrist University Hospital, Faculty of Medicine, University of Zurich, Zurich, Switzerland; 2 Swiss Center for Musculoskeletal Imaging, Balgrist Campus AG, Zurich, Switzerland; 3 Department of Orthopedics, Balgrist University Hospital, Faculty of Medicine, University of Zurich, Zurich, Switzerland

**Keywords:** Coccydynia, Coccyx, MRI, CT, Pain assessment

## Abstract

**Objectives:**

To analyze pain relief for chronic coccydynia after CT-guided, pericoccygeal ropivacaine and triamcinolone injection over time and to investigate possible imaging predictors of successful and maintained pain relief.

**Materials and methods:**

In this retrospective study, chronic coccydynia patients receiving CT-guided pericoccygeal injections with ropivacaine and triamcinolone were assessed for pain relief post-injection, using a numeric rating scale (NRS, 0–10) and percentage pain reduction (PPR, %). Patients were followed up until pain recurrence. Two radiologists assessed MRI findings prior to, and contrast distribution during CT injection. Imaging predictors for successful pain relief at 1 month (PPR ≥ 50%) combined with maintained pain relief for at least 3 months post-injection were investigated using logistic regression analysis.

**Results:**

63 patients (mean age 44.6 ± 12.5 years) were evaluated. Average pain dropped from initially 6.3 to 3.0 (average PPR 53.2 ± 37.5%) 15 min post-injection, and to 2.9 (average PPR 55.2 ± 41.4%) one month post-injection (all *p* < 0.001). One month post-injection, 41 patients (65%) reported PPR ≥ 50%. Pain relief persisted on average for 6 months. Three months post-injection, 32/63 patients (50.8%) reported maintained pain relief. Fluid-sensitive sequence (FSS)-hyperintensity of the sacro-/intercoccygeal synchondrosis, coccyx subluxation and a bony spicule were the only significant predictors (odds ratios: 3.5–5.1) of successful and maintained pain relief (all *p* ≤ 0.031).

**Conclusion:**

CT-guided pericoccygeal injection with ropivacaine and triamcinolone allowed successful pain relief in 65% of chronic coccydynia patients, with a PPR of 55% and persistence of 26 weeks on average. Odds of successful and maintained pain relief increased with the presence of a bony spicule, subluxation and FSS-hyperintensity of the synchondrosis.

**Key Points:**

***Question*** *Imaging predictors of successful pain relief in chronic coccydynia patients receiving pericoccygeal ropivacaine and triamcinolone injections are unknown.*

***Findings*** *A bony spicule, subluxation, and sacro-/intercoccygeal fluid-sensitive sequence hyperintensity on MRI were significant predictors of successful pain relief for ≥ 3 months. CT-contrast distribution did not influence pain relief.*

***Clinical relevance*** *CT-guided pericoccygeal injections with ropivacaine and triamcinolone offer successful pain relief in chronic coccydynia; the presence of a bony spicule, subluxation, and sacro-/intercoccygeal fluid-sensitive sequence hyperintensity was predictive of pain relief.*

**Graphical Abstract:**

**Figure:**
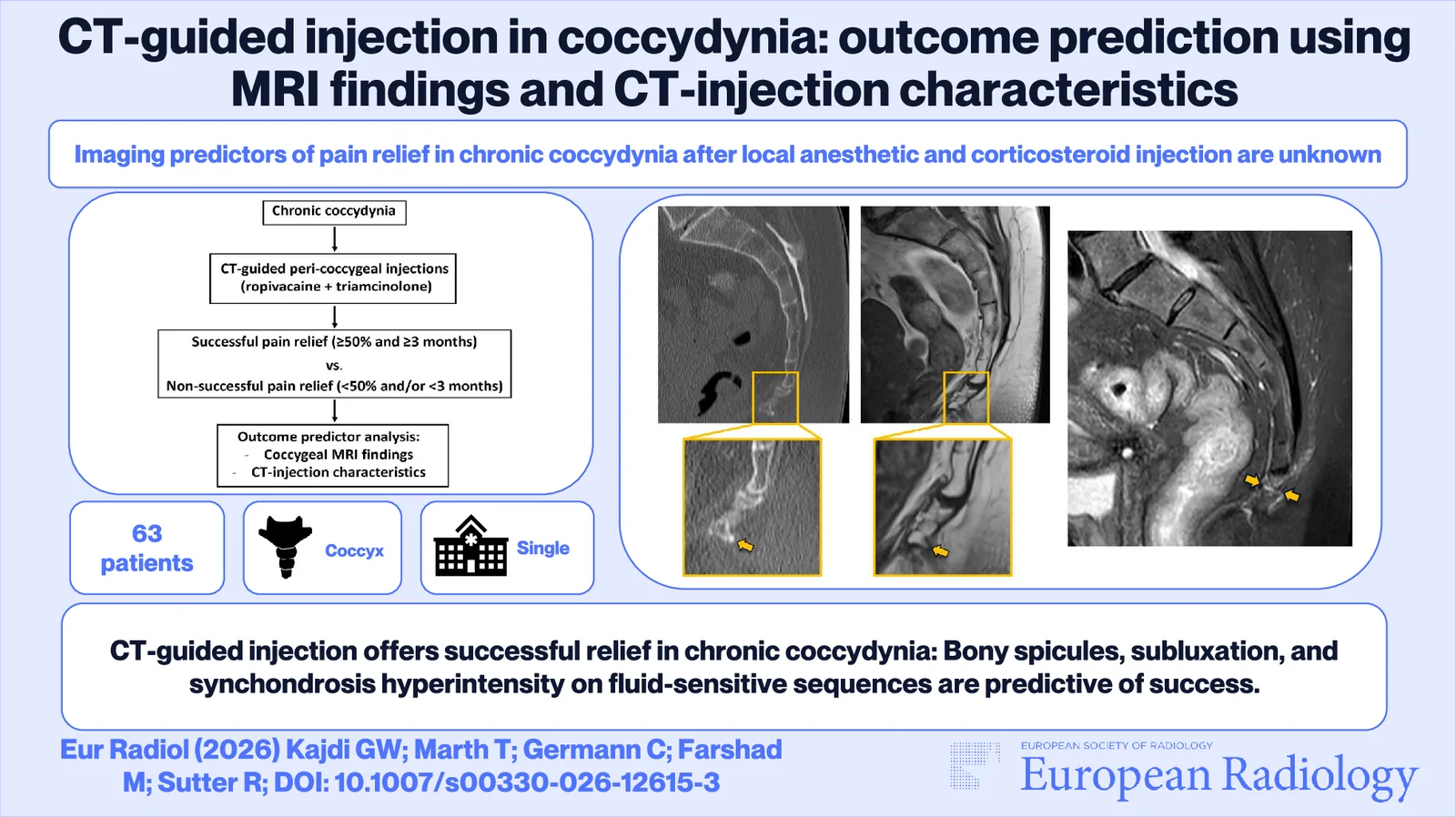

## Introduction

Lower back pain (LBP) is a medically and economically important disease, which is a major reason for disability in the age group < 45 years of age [1]. Coccydynia, describing pain in the tailbone, accounts for 1–3% of these cases [2]. It is a complex clinical condition that may occur with or without identifiable imaging abnormalities [3, 4], and the prognostic impact of such findings on treatment outcome remains unclear.

Combined local anesthetic and corticosteroid injections are safe, and they are an important diagnostic and treatment tool in coccydynia [5, 6], either after failure of, or in conjunction with other conservative treatments such as physical therapy, stool softeners, cushioning while sitting, and non-steroidal anti-inflammatory drugs (NSAIDs) [7, 8]. Surgical resection of the tailbone (coccygectomy) is generally deemed undesirable, due to high complication rates, with up to 22% showing post-operative wound infection [7], and is therefore only used in refractory or chronic cases as a last resort of therapy [9].

Imaging plays a crucial role in the diagnostic and treatment work-up of coccydynia: Conventional radiographs may be used to exclude acute fractures and assess coccygeal mobility in selected cases [10], however due to their limited diagnostic yield, nowadays reliance on more advanced imaging techniques is favored: MRI can reveal the anatomic origin and other correlates of pain such as degenerative, inflammatory, post-traumatic and/or neoplastic changes of the coccyx and its surrounding tissues [11, 12]. For pain treatment, ultrasound [13], fluoroscopy [14], and CT guidance [15] facilitate focused navigation of the injection to the suspected pain origin in the coccyx.

Despite knowledge of these possible pain correlates on imaging [12], especially MRI, and frequently employed CT guidance [15] for coccyx pain injection, no study has evaluated imaging findings in these modalities as possible predictors for pain relief after pain injection.

The purpose of this study was therefore to evaluate the magnitude and duration of pain reduction after combined local anesthetic and corticosteroid injections to the coccyx in patients with chronic coccydynia, to assess potential imaging outcome predictors based on prior MRI findings, CT injection target and CT contrast distribution during the injection.

## Materials and methods

### Patient selection

This retrospective single-center study was approved by the institutional review board and carried out according to the principles of the Declaration of Helsinki, as well as national ethical standards. All patients included in the study gave informed consent, allowing their health-related data to be used for research.

Our picture archiving and communication system (PACS) and clinical information system were reviewed for LBP patients ≥ 18 years of age, with chronic coccydynia (≥ 3 months) refractory to conservative therapy alone, who had received CT-guided (sacro-) coccygeal injections with ropivacaine and triamcinolone between August 2015 and October 2024. This resulted in 146 potentially eligible patients. Patients with sacral or coccygeal tumors were not included, as tumor-related pain patients follow a different diagnostic and therapeutic pathway in our institution.

**Fig. 1:**
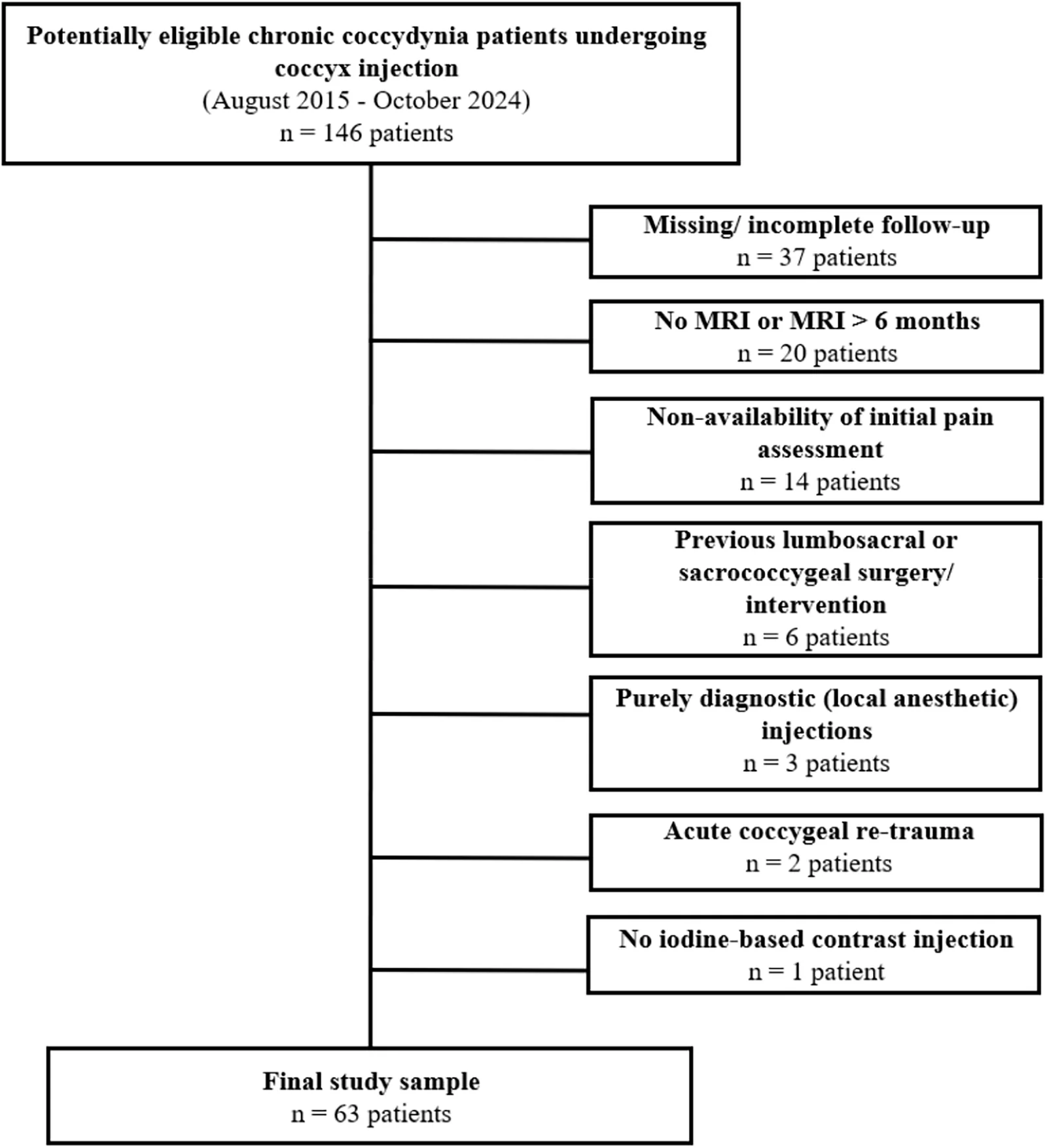
Flowchart of the patient selection process for patients who received CT-guided injection with ropivacaine and triamcinolone. From *n* = 146 potentially eligible patients, 83 patients had to be excluded during the selection process, resulting in a final study sample of 63 patients with a coccyx injection

Exclusion criteria included (1) missing or incomplete follow-up (*n* = 37), (2) missing pre-injection MRI or MRI > 6 months prior to coccyx injection (*n* = 20), (3) non-availability of initial pain evaluation (*n* = 14), (4) previous lumbosacral or sacrococcygeal surgery/intervention (*n* = 6), (5) purely diagnostic injections with local anesthetic (*n* = 3), (6) acute on chronic disease with re-trauma (≤ 3 months) of the coccyx (*n* = 2), and (7) coccyx injection without iodine-based contrast agent due to possible prior allergic reaction (*n* = 1). A total of 63 patients were included in the study (Fig. 1).

### Magnetic resonance imaging

Combined MRI of the sacrum and coccyx was conducted on either a 1.5-T unit (Magnetom Avanto Fit and Magnetom Sola, Siemens Healthineers) (*n* = 30 patients, 47.6%) or a 3-T unit (Magnetom Vida and Magnetom Skyra Fit, Siemens Healthineers) (*n* = 33 patients, 56.3%) using a 32-channel phased-array spine coil. All patients underwent routine combined sacrum and coccyx imaging protocols in supine and feet-first position, without MR-contrast administration. The sequence parameters varied depending on the scanner (1.5 T vs. 3 T) (Supplementary Table S1).

### Image analysis

Image analysis was performed using a commercially available picture archiving and communication system (PACS) workstation (Merlin, Phoenix-PACS). All CT and MRI studies were anonymized and independently reviewed by two board-certified and musculoskeletal fellowship-trained radiologists (G.W.K. and T.M., with 8 and 4 years of experience, respectively). Examinations were analyzed in random order, whereby both readers were blinded to clinical data.

**Fig. 2:**
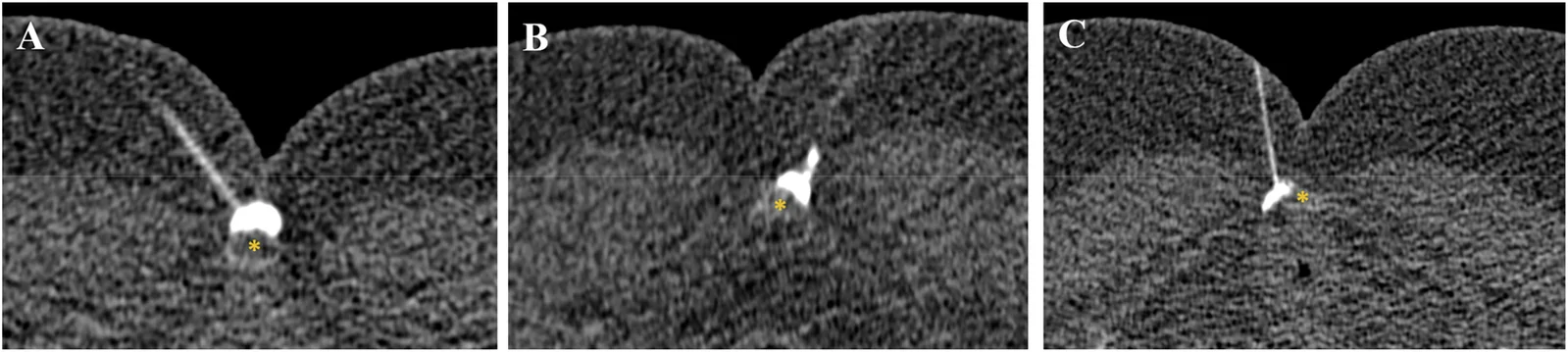
Three different contrast distribution patterns during CT-guided coccyx pain injections in three different patients. Pericoccygeal contrast distribution within the soft tissues immediately surrounding the coccyx was purely extra-retroperitoneal in our study. We differentiated between posterior (**A**), posterolateral (**B**) and intraligament (**C**) contrast distribution. Axial CT images are shown, with all patients lying in a prone position, and the needle inserted from a slightly lateral position. The coccyx is illustrated with an orange asterisk

For each CT scan, readers indicated the contrast distribution pattern during pain injection as either immediately posterior to the coccyx, posterolateral to the coccyx or as intra-ligament contrast distribution (Fig. 2).

**Fig. 3:**
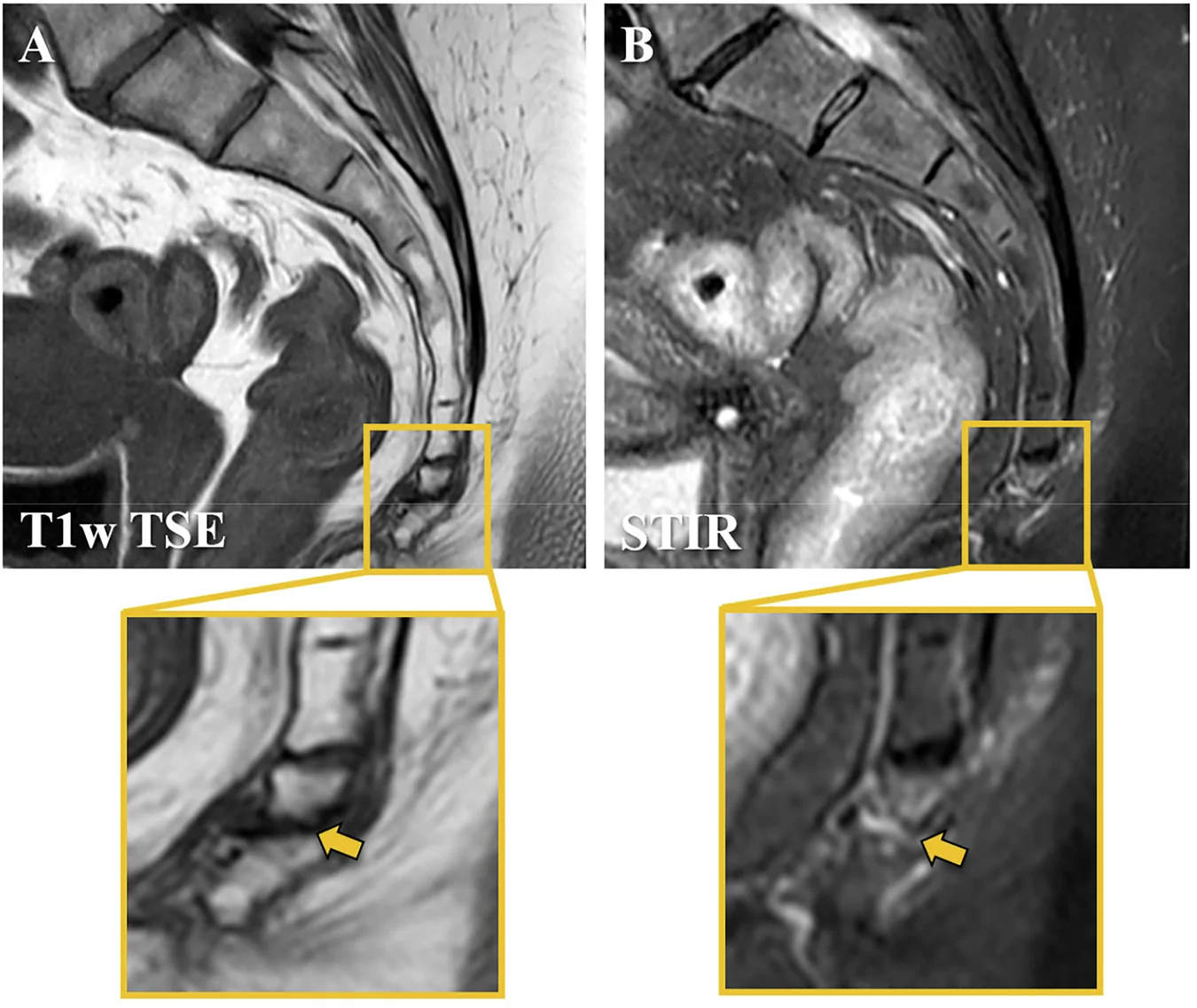
MRI findings in a 54-year-old female chronic coccydynia patient with successful pain relief (PPR = 80%) and maintained pain relief (15 weeks) following CT-guided coccyx injection. The magnified insets (orange outline, **A**, **B**) focus on the Co2/3 segment (orange arrows). Both the sagittal T1w image (**A**) and the sagittal FSS (here: sagittal STIR image) (**B**) show cortical endplate irregularities along the Co2/3 synchondrosis, with additional detection of endplate edema and a clear linear FSS-hyperintensity within the Co2/3 synchondrosis. Co, coccygeal; FSS, fluid-sensitive sequence; PPR, percentage pain relief; STIR, short tau inversion recovery; TSE, turbo spin echo

**Fig. 4:**
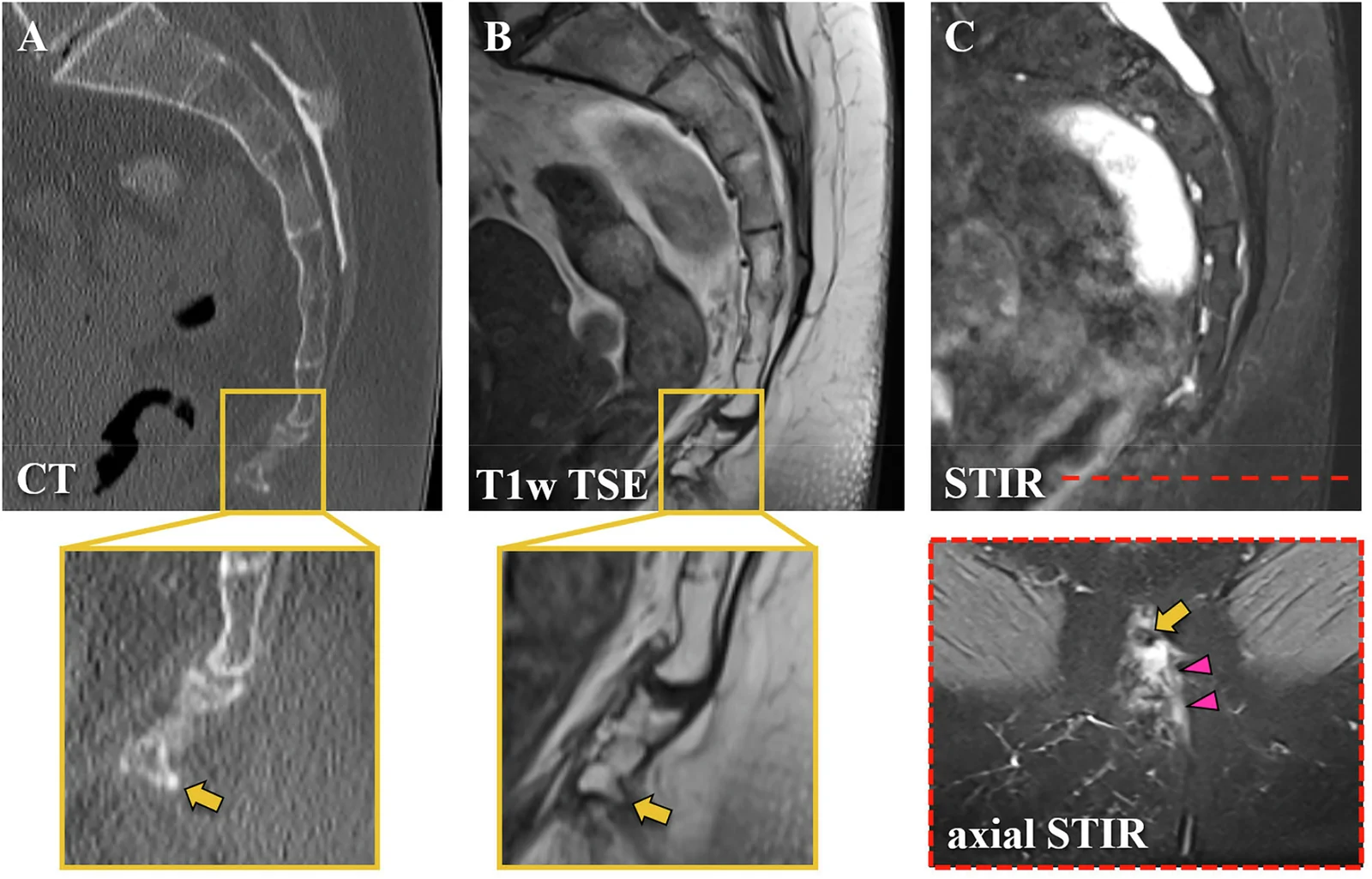
Bony spicule in a 63-year-old female chronic coccydynia patient, with successful pain relief (PPR = 100%) and maintained pain relief (52 weeks) following CT-guided coccyx injection. A posteroinferiorly angled bony spicule of the coccygeal apex is illustrated (orange arrows) on both the sagittal CT image (**A**, used for illustrative purposes only) and MRI (**B**, sagittal T1w image). While the bony spicule and its associated soft-tissue edema are obscured on the strictly midline sagittal FSS (here sagittal STIR image) (**C**, upper image), both the spicule (**C**, orange arrow) and the adjacent posterolateral soft-tissue edema (**C**, pink arrowheads) are well depicted on the axial FSS (here axial STIR image) (**C**, red dotted line). TSE, turbo spin echo; FSS, fluid-sensitive sequence; STIR, short tau inversion recovery

On MRI, readers assessed potential pain correlates along the coccyx: At the sacrococcygeal and intercoccygeal junctions the presence of Modic type I changes [11, 16] (yes or no), endplate irregularities and spondylophytes (yes or no) [11], and fluid-sensitive sequence (FSS)-hyperintensity within the sacro-/interecoccygeal synchondrosis [11, 12, 17] (yes or no) were evaluated (Fig. 3). Modic type I changes/bone marrow edema were assessed qualitatively and categorized as present or absent. Only clearly demarcated hyperintense areas on fluid-sensitive sequences not compatible with linear vascular structures were considered edema. Degenerative changes such as osteophytes were recorded only when clearly visible on MRI. Subtle osteophytes detected exclusively on CT were not considered. Furthermore, coccyx morphology according to Postacchini and Massobrio [18, 19], sacrococcygeal fusion (yes or no) [20], and intercoccygeal fusion (yes or no) [12, 20], the sacrococcygeal and intercoccygeal slope (in degrees) [12], sacrococcygeal and/or intercoccygeal (sub-) luxation (yes or no) [11, 12], and presence of a posterior bony spicule [11, 12] in the last coccygeal segment (yes or no) were assessed (Fig. 4). Presence of soft-tissue edema and/or bursitis around the coccyx (yes or no) [11, 12] (yes or no) [12] were also recorded. If no sacrococcygeal abnormalities were detected on MRI, the patient was considered imaging-negative and recorded as such.

### CT-guided coccygeal pain injections

All coccygeal pain injections were performed by board-certified, fellowship-trained musculoskeletal radiologists, with 5–19 years of experience. The injection protocol was standardized to ensure intervention consistency. The site of the injection was chosen jointly by the treating radiologist and referring orthopedic surgeon, considering the localization of the most extensive pathologic changes on the previous MRI. Injection sites were categorized as targeting either the sacro-/intercoccygeal synchondrosis or the coccygeal apex. Synchondrosis-targeted injections were retrospectively further subclassified as performed directly at the synchondrosis level or immediately adjacent to it along the cortical bone in a periosteal approach. Injections were guided by a 64- or 128-detector row CT (Philips Brilliance, Philips Medical Systems; SOMATOM Definition AS, SOMATOM Definition AS+, or SOMATOM Edge Plus, Siemens Healthineers). Patients lay still in a prone position during the procedure. CT imaging was acquired over the entire coccyx from its base, including the sacrococcygeal junction, down to the coccygeal apex. The best access for the needle (20 G, 7 cm or 21 G, 12 cm, Braun) to the injection target was chosen by the radiologist. After skin disinfection and subcutaneous application of local anesthetic, the needle was introduced under CT guidance from posterolateral until its tip reached the snychondrosis, the posterior to posterolateral cortical bone adjacent to the synchondrosis or the coccyx apex (Fig. 2). An injection of 0.5 mL iopamidol (Iopamiro 300, 300 mg of iodine per milliliter; Bracco) was performed to verify correct needle tip positioning. After contrast agent injection, 1 mL (40 mg/mL) crystalline triamcinolone (Triamcort Depot; Zentiva) was slowly injected, followed by a slow injection of 1 mL of 0.2% ropivacaine (Naropin; Astra-Zeneca).

### Pain evaluation: magnitude and duration

Pain levels were assessed right before the pain injection (baseline), 15 min after, and at follow-up 1 month (4–5 weeks) post-procedure (either in clinical consultation or by phone consultation). For assessment, the numerical rating scale (NRS) was used, where 0 indicated no pain, and 10 indicated the worst pain imaginable. Percent change in NRS was calculated from pre-procedure to 15 min and to 1 month post-procedure. The percentage of pain reduction 1 month post-procedure was used to evaluate the categorical outcome of successful pain relief: Based on previous literature, the threshold for successful pain relief was set at ≥ 50% pain reduction prior to data analysis [21, 22]. All other responses were considered as not improved for our analysis. All patients included in the study gave notice to the orthopedic outpatient clinic of our hospital (either by phone or by in-person visit) after the pain injection, when the symptoms of coccydynia were subjectively not sufficiently suppressed anymore or had reappeared.

### Statistical analysis

All statistical analyses were performed in SPSS Statistics (v. 29, IBM Corporation).

Normal and non-normal distribution of data was assessed graphically and analytically using Quantile-Quantile plots and the Shapiro–Wilk test. With the primary outcome being successful and combined maintained (≥ 3 months) pain relief, analyzed as a binary variable (yes/no), responders and non-responders were initially compared using Fisher’s exact test for dichotomous variables, the Fisher–Freeman–Halton test for categorical variables with more than two categories, and the Mann–Whitney U-test for continuous variables. Additionally, binary logistic regression was used throughout the analysis. First, an exploratory univariable binary logistic regression was performed to evaluate associations between individual clinical variables/potential confounders (age, sex, BMI, injector experience), predefined MRI findings with documented evidence of pain-correlation in the literature, injection target characteristics, and the outcome. This exploratory step was intended for descriptive purposes and hypothesis generation. Subsequently, analyses focused on a limited number of clinically plausible primary imaging predictors. To assess the robustness of these associations, adjusted sensitivity analyses were performed using binary logistic regression, with separate multivariate models adjusted individually for age, sex, BMI and injector experience. These covariates were not entered simultaneously in order to avoid model overfitting, given the limited number of outcome events (*n* = 32). Inter-reader agreement was analyzed by kappa statistics (Cohen’s κ) for all categorical outcomes and intraclass correlation coefficients (ICC) for all continuous numeric outcomes. The level of agreement for kappa statistics was categorized as follows [23]: 0.0 = poor, 0.01–0.20 slight, 0.21–0.40 = fair, 0.41–0.60 = moderate, 0.61–0.80 = substantial, 0.81–1.00 = almost perfect agreement. ICC was categorized as follows: 0.0–0.49 = poor, 0.5–0.74 = moderate, 0.75–0.89 = good, 0.9–1.0 = excellent agreement. All statistical tests were performed two-sided, and a level of significance (α) of 0.05 was used.

## Results

### Patients

63 patients (mean age 44.6 ± 12.5 years, 40 females) were included in the study. All of them had received a prior coccyx MRI on average 4.5 weeks (range: 0–26) prior to the CT-guided injection. Detailed demographic data is given in Table 1. Patient age, BMI, as well as chronicity, etiology of coccydynia and patient sex did not show significant correlation to sufficient pain (PPR ≥ 50%) and maintained pain relief ≥ 3 months (all *p* ≥ 0.21).

**Table 1:** Patient demographics (*n* = 63) and pain improvement

	Mean ± SD or *n* (%)
Age (years)	44.6 ± 12.5
Females (%)	40 (63.5)
BMI (kg/m^2^)	26.6 ± 5.2
Chronicity (in months)	28.6 ± 36.7
Etiology	
Traumatic	32 (50.8)
Non-traumatic	31 (49.2)
Injection site	
Direct synchondrosis level	20 (31.7)
Synchondrosis adjacent cortical bone	38 (60.3)
Coccyx apex	5 (7.9)
Pain (NRS, 0–10)	
Before injection	6.3 ± 2.2
15 min after injection	3.0 ± 2.5
1 month after injection	2.9 ± 2.9
Pain % change	
15 min after injection	−53.2 ± 37.5
1 month after injection	−55.2 ± 41.4
Pain relief persistence (in weeks)	25.3 ± 31.4

*NRS* numeric rating scale, *SD* standard deviation

### Imaging findings and procedural variables in responders vs. non-responders

In Table 2, the absolute number and percentage of patients with all assessed MR imaging findings are summarized for both readers. Injection target site stratification and analysis in relation to the predefined MRI findings and treatment outcome are summarized in Table 3. Comparing MRI findings and CT procedural variables between responders and non-responders (Table 4) showed a significantly higher prevalence of FSS-hyperintensity of the synchondrosis (*p* = 0.023), of sacro-/intercoccygeal subluxation (*p* = 0.027), and presence of a posterior bony spicule at the coccyx apex (*p* = 0.014) among responders. No other significant subgroup differences were found. These findings aligned with exploratory univariable binary logistic regression analyses (Supplementary Table S2), which likewise demonstrated significant (all *p* ≤ 0.02) associations between treatment success and FSS-hyperintensity of the synchondrosis (OR: 3.6; CI: 1.3–10.2), sacro-/intercoccygeal subluxation (OR: 4.0; 1.2–13.2), and presence of a posterior bony spicule (OR: 4.6; 1.5–15). Despite relatively wide confidence intervals, reflective of the limited number of outcome events (*n* = 32), the observed odds ratios remained stable among the final multivariable binary logistic regression analyses adjusted individually for age, sex, BMI, and injector experience (Table 5).

**Table 2:** Proportion of imaging findings by reader (*n* = 63), and inter-reader reliability

Imaging finding, *n* (%)	Reader 1	Reader 2	κ/ICC (95% CI)
**CT**			
Contrast distribution			0.95 (0.87–0.99)
Posterior	23 (36.5)	21 (33.3)	
Posterolateral	29 (46.0)	29 (46.0)	
Lateral (intra-ligament)	11 (17.5)	13 (20.6)	
**MRI**			
Modic Type I changes			0.80 (0.70–0.90)
Present	29 (46)	23 (36.5)	
Not present	34 (54)	40 (63.5)	
Endplate irregularities and spondylophytes			0.71 (0.51–0.91)
Present	48 (76.2)	45 (71.4)	
Not present	15 (23.8)	18 (28.6)	
Sacro-/intercoccygeal FSS-hyperintensity			0.94 (0.84–0.99)
Present	28 (44.4)	28 (44.4)	
Not present	35 (55.6)	35 (55.6)	
Postacchini classification (coccyx morphology)			0.80 (0.66–0.94)
Type 1	25 (39.7)	23 (36.5)	
Type 2	21 (33.3)	23 (36.5)	
Type 3	5 (7.9)	5 (7.9)	
Type 4	7 (11.1)	7 (11.1)	
Type 5	2 (3.2)	2 (3.2)	
Type 6	3 (4.8)	3 (4.8)	
Sacro-/intercoccygeal subluxation			0.96 (0.88–0.99)
Present	19 (30.2)	18 (28.6)	
Not present	44 (69.8)	45 (71.4)	
Sacrococcygeal fusion			0.97 (0.89–0.99)
Present	21 (33.3)	22 (34.9)	
Not present	42 (66.7)	41 (65.1)	
Intercoccygeal fusion			0.96 (0.88–0.99)
Present	48 (76.2)	49 (77.8)	
Not present	15 (23.8)	14 (22.2)	
Sacrococcygeal angle	112.4 ± 15.0	112.4 ± 15.2	0.99 (0.98–0.99)
Intercoccygeal angle	135.4 ± 25.5	135.0 ± 24.8	0.98 (0.97–0.99)
Posterior bony spicule			0.89 (0.77–0.99)
Present	20 (31.7)	21 (33.3)	
Not present	43 (68.3)	42 (66.7)	
Soft-tissue edema and/or bursitis			0.94 (0.86–0.99)
Present	32 (50.8)	32 (50.8)	
Not present	31 (49.2)	31 (49.2)	

*CI* confidence interval, *FSS* fluid-sesitive sequence, *ICC* intraclass correlation coefficient, *κ* Cohen’s Kappa

**Table 3:** Distribution of injection targets and treatment success across MRI-defined diagnostic subgroups

	*n*	Direct synchondrosis level, *n* (%)	Synchondrosis adjacent cortical bone, *n* (%)	Coccyx apex, *n* (%)	Treatment success, *n* (%)
Modic Type I changes	29	11 (37.9)	16 (55.2)	2 (6.9)	18 (62.1)
Endplate irregularities and spondylophytes	48	19 (39.6)	26 (54.2)	3 (6.3)	27 (56.3)
FSS-hyperintensity of the synchondrosis	28	9 (32.1)	17 (60.7)	2 (7.1)	19 (67.9)
Posterior bony spicule	20	5 (25.0)	10 (50.0)	5 (25.0)	17 (85.0)
Subluxation	18	10 (55.6)	6 (33.3)	2 (11.1)	19 (78.9)
Soft-tissue edema/bursitis	32	11 (34.4)	18 (56.3)	3 (9.4)	21 (65.6)
Imaging-negative	5	1 (20.0)	4 (80.0)	0 (0.0)	1 (20.0)
Overall	63	20 (31.7)	38 (60.3)	5 (7.9)	32 (50.8)

**Table 4:** Comparison of MRI findings and procedural variables between responders and non-responders

MRI finding	Responders (*n* = 32)	Non-responders (*n* = 31)	*p*-value*
Modic Type I changes	18 (56.3%)	11 (35.5%)	0.13
Endplate irregularities and spondylophytes	27 (84.4%)	21 (67.7%)	0.15
Sacro-/intercoccygeal FSS-hyperintensity	19 (59.4%)	9 (29.0%)	**0.023**
Postacchini classification (coccyx morphology)			0.06
Type I	14 (43.8%)	11 (35.5%)	
Type II	8 (25.0%)	13 (41.9%)	
Type III	1 (3.1%)	4 (12.9%)	
Type IV	7 (21.9%)	0 (0.0)	
Type V	1 (3.1%)	1 (3.2%)	
Type VI	1 (3.1%)	2 (6.5%)	
Sacro-/intercoccygeal subluxation	14 (43.8%)	5 (16.1%)	**0.027**
Sacrococcygeal fusion	14 (43.8%)	7 (22.6%)	0.11
Intercoccygeal fusion	25 (78.1%)	23 (74.2%)	0.77
Sacrococcygeal angle (mean ± SD)	113.6 ± 15.1	111.2 ± 14.9	0.52
Intercoccygeal angle (mean ± SD)	135.9 ± 25.5	135.0 ± 25.5	0.97
Posterior bony spicule	15 (46.9%)	5 (16.1%)	**0.014**
Soft-tissue edema/bursitis	19 (59.4%)	13 (41.9%)	0.21
Imaging-negative	1 (3.1%)	4 (12.9%)	0.20
Injection target sites			0.50
Direct synchondrosis level	10 (31.3%)	10 (32.3%)	
Synchondrosis adjacent cortical bone	18 (56.3%)	20 (62.5%)	
Coccyx apex	4 (12.5%)	1 (3.2%)	
CT-contrast distribution pattern			0.63
Posterior	12 (37.5%)	11 (35.5%)	
Posterolateral	16 (50.0%)	13 (41.9%)	
Intra-ligament	4 (12.5%)	7 (22.6%)	

*FSS* fluid-sensitive sequence, *SD* standard deviation

* Continuous variables were compared between independent groups using the Mann–Whitney U-test, binary categorical variables were compared using Fisher’s exact test, and multivariable categorical variables were compared using Fisher–Freeman–Halton exact test

Bolded *p*-values signify signifi cant diff erences between responders and non-responders

**Table 5:** Adjusted regression models of selected MR imaging findings and treatment success

Imaging finding	Unadjusted	Age adjusted	Sex adjusted	BMI adjusted	Injector experience adjusted
FSS-hyperintensity at synchondrosis	3.6 (1.3–10.2)	4.1 (1.3–12.5)	4.4 (1.4–13.4)	3.9 (1.3–11.8)	3.5 (1.2–10.1)
Subluxation/instability	4.0 (1.2–13.2)	4.0 (1.2–13.2)	4.3 (1.3–14.5)	4.7 (1.3–17.2)	4.0 (1.2–13.3)
Posterior bony spicule	4.6 (1.4–14.9)	4.9 (1.5–16.5)	4.1 (1.2–13.9)	5.1 (1.5–17.3)	4.6 (1.4–15.0)

Odds ratios (ORs) with 95% confidence intervals (CIs) were derived from separate binary logistic regression models. Unadjusted models included only the respective MRI finding. Adjusted analyses were performed by adding age, sex, BMI and injector experience individually to avoid overfitting, given the limited number of outcome events

Among patients with FSS-hyperintensity of the synchondrosis, 77% achieved successful pain relief and 69% maintained pain relief for ≥ 3 months. Corresponding rates were 84% and 74% in patients with subluxation, and 86% and 76% in patients with a posterior bony spicule.

### Magnitude of pain response at 15 min and 1 month post-injection

Average pain assessment on the NRS (0–10) was 6.3 ± 2.2 at pre-injection baseline, 3.0 ± 2.5 immediately (15 min) post-injection, and 2.9 ± 2.9 at the 1-month (4–5 weeks) follow-up. Average percentage pain relief from baseline NRS was 53.2% ± 37.5% immediately post-injection (*p* < 0.001) and 55.2% ± 41.4% at the 1-month follow-up (*p* < 0.001) (Table 1).

Supplementary Table S3 offers an overview of the proportion of responders (PPR ≥ 50%) and non-responders (PPR ≥ 50%).

### Duration of pain response

On average, patients reported recurrence of insufficiently suppressed coccydynia at 6 months post-injection (25.3 ± 31.4 weeks): Ten of the 63 included patients (16%) did not have any pain relief at any given time-point after the injection. Of the seven patients (11%) with pain relief for less than a month, 4/63 (6%) showed maintained pain relief for ≤ 1 week, and 3/63 (5%) showed maintained pain relief ≥ 2 weeks and < 4 weeks. The remaining 46/63 patients (73%) reported maintained pain relief at 4 weeks, with 41/46 (89%) having a PPR ≥ 50%. After 3 months, 32/63 patients (50.8%), after 6 months, 24/63 patients (38.1%), after 9 months, 18/63 (28.6%), after 12 months, 12/63 (19%), after 15 months, 7/63 maintained subjectively sufficient pain relief, after 18 months, 4/63, after 21 months, 3/63. By 18 months, > 90% of the study population had reported recurrence of insufficiently suppressed coccydynia.

Supplementary Table S3 offers an overview of the proportion of responders (maintained pain relief ≥ 3 months) and non-responders (maintained pain relief < 3 months).

## Discussion

In this study, we demonstrated that CT-guided injections with ropivacaine and triamcinolone allow for successful pain relief in 65% of chronic coccydynia patients after 1 month, with an average percentage pain relief (PPR) of 55%. Half of all patients experienced maintained pain relief for at least 3 months. The odds of sufficient and maintained pain relief increased three- to five-fold when a posterior bony spicule was present on the coccyx, with subluxation or when FSS-hyperintensity of the sacro- or intercoccygeal synchondrosis was seen. As of today, our study comprises the only cohort for which successful and maintained pain relief after CT-guided coccyx injection has been predicted based on previous MRI findings.

Both obesity and female sex are known predisposing factors for coccydynia, regardless of traumatic or non-traumatic origin [8, 24]. That is consistent with our study’s demographic data, with 60% of affected patients being women and having a slightly elevated body mass index (BMI) with an average of 27 kg/m^2^, as well as equal distribution between traumatic and non-traumatic cases.

Previous research on interventional treatment of coccydynia focused on non-image- [6], as well as image-guided injection approaches for pain treatment [13, 15, 25]. The latter often aim for the sacrococcygeal/intercoccygeal disc space, or for the retroperitoneal ganglion impar (GI) [15, 25]. Our study sets itself apart from previous studies regarding the injection approach, with proof of solely pericoccygeal soft-tissue contrast distribution and no intradiscal or retroperitoneal spread, despite frequent needle tip positioning along the synchondrosis. A pericoccygeal soft-tissue distribution pattern has been hypothesized to be the main type of injection contrast distribution [2] when aiming for the disc space, but this has not been explicitly demonstrated until now. Different contrast distribution patterns (e.g., posterior, posterolateral, or intraligament) within the pericoccygeal soft tissues were not predictive of pain relief in our study at any studied time point. When compared to the literature on the established ganglion impar block (GiB), sufficient (*n* = 41; 65%) and maintained pain relief (*n* = 32; 51%) was still readily achievable with pericoccygeal contrast distribution. With a 55% PPR on average 1 month post-injection, pericoccygeal pain injection was comparable and even slightly superior in effect to GiB outcomes, recently reported by Kaya et al (*n* = 26) [14] (1 month PPR of 53%), and Nasiri et al (*n* = 26) (1 month PPR of 44%). Contrasting the established GiB method, our approach was less invasive without the need for trans-gluteal, trans-coccygeal, or sacrococcygeal injection of the retroperitoneum, which can have very rare but severe complications, such as transient motor, bowel, bladder and sexual dysfunction, and even rectal perforation [26–29]. As the injection site was selected according to the presumed MRI pain correlated, a potential influence of target choice on outcome cannot be excluded. In our exploratory analysis, however, no consistent target-dependent effect was observed, suggesting that treatment response may be driven more by the presence of a relevant structural correlate than by the exact injection site.

In our cohort, successful and maintained pain relief was achieved in approximately half of the refractory patients, leaving a substantial proportion with persistent symptoms. For these individuals, escalation strategies such as radiofrequency-based intervention [30, 31] may represent a less invasive alternative to surgery, while successful neuromodulation [32] and oxygen-ozone therapy [33, 34] have also been described, albeit with currently limited evidence in coccydynia.

Beyond the injection approach itself, the choice of imaging guidance also warrants discussion. Ultrasound [13] and CT guidance [15] represent two accurate alternatives for coccygeal injection. Ultrasound precision may be more operator-dependent, whereas CT provides high anatomical accuracy at the expense of additional ionizing radiation, making the choice of guidance one based on local expertise and clinical context, offering potential for future comparative studies. Of all assessed MR imaging findings previously shown to be associated with coccydynia [12] and included in this study, only FSS-hyperintense signal within sacro- or intercoccygeal disc, coccygeal subluxation and a posterior bony spicule of the tailbone apex were shown to be significant predictors of successful and maintained pain relief. This implies a more causative relationship between these imaging findings and coccydynia when compared to all other assessed potential predictors.

A bony spicule is an intuitive pain generator, due to its rigidity, creating a non-yielding surface against which the displaceable surrounding soft tissues can rub, causing inflammation and pain [11, 12, 35]. Our results on bony spicules as a promising pain relief predictor align with findings by Doursounian et al (*n* = 33) [36]: They reported a success rate of about 80% for coccygectomy in chronic refractory coccydynia patients with bony spicules (*n* = 33). Thus, these multi-study observations should encourage radiologists to explicitly report this finding on coccyx MRI. It further raises the question if less invasive surgical approaches, such as partial coccygectomy [37], might be a better surgical fit for this specific coccydynia-with-spicule cohort, rather than the complication-ridden [7] but established last resort of total coccygectomy.

Based on histopathologic correlation by Balain et al [17], FSS-hyperintensity within the sacrococcygeal or intercoccygeal synchondrosis is similar in nature to the disc changes observable in spine degeneration due to altered spine mobility [38], ranging from disc dissecation to disc effusion and even liquefication [39]. Fitting this analogy, coccydynia is known to be associated with hyper- and hypomobility of the coccygeal segments [40, 41]. Although hypermobility was not explicitly examined in our study, subluxation, a static image representation of segmental hypermobility [11, 12, 41], also significantly increased the odds of successful and maintained pain relief. Disc or synchondrosis FSS-hyperintensity as a potential, degenerative sequelae of altered segmental mobility, is also known to be a possible pain generator [42–44], fitting with our observations.

Only 7.9% of patients presented with inconspicuous MRI findings, likely reflecting selection bias toward cases with identifiable imaging correlates. Interestingly, treatment success in this imaging-negative subgroup (20%) was markedly lower than the overall cohort average (50.8%), suggesting limited benefit of infiltration in the absence of structural abnormalities.

Limitations of this study need to be addressed. Associations observed should be interpreted in light of the limited sample size (*n* = 63) and number of outcome events (*n* = 32), which constrained model complexity and necessitated a parsimonious analytical approach. Nevertheless, adjusted sensitivity analyses accounting individually for patient sex, age, BMI, and injector experience yielded comparable results, supporting the robustness of the main findings. The absence of a randomized control group precludes conclusions regarding comparative efficacy. Although cohort heterogeneity was reduced by restricting inclusion to patients with chronic coccydynia refractory to prior physiotherapy and oral analgesic treatment, continued physiotherapy and concomitant use of oral pain medication after injection were not systematically documented and may represent residual confounding. In addition, strict inclusion criteria requiring complete baseline and follow-up documentation resulted in the exclusion of a substantial number of potentially eligible patients, which may have introduced selection bias, with imaging-negative patients and patients without pain recurrence likely underrepresented. Some treatment target subgroups, particularly apex-targeted injections, were small, limiting target-specific analyses. Imaging relied on static MRI and CT; coccygeal mobility could therefore not be directly assessed. Moreover, follow-up MRI was not routinely performed after injection, precluding assessment of post-treatment changes in hyperintensity on FSS-sequences.

In conclusion, this study revealed that CT-guided pericoccygeal injections with ropivacaine and triamcinolone allowed for sufficient pain relief in 65% of chronic coccydynia patients, with a percentage pain relief of 55%, persisting for 26 weeks on average. The odds of sufficient and maintained pain relief significantly increased in the presence of bony spicules, subluxation and FSS-hyperintensity at the synchondrosis.

## Supplementary information

Supplementary information

## Abbreviations

CI: Confidence interval
FSS: Fluid-sensitive sequence
GiB: Ganglion impar block
ICC: Intraclass correlation coefficient
LBP: Lower back pain
NRS: Numeric rating scale
OR: Odds ratio
PPR: Percentage pain relief
STIR: Short tau inversion recovery
TSE: Turbo spin echo
