# Supplementary material for: CT-guided injection in coccydynia: outcome prediction using MRI findings and CT-injection characteristics
Source: Eur Radiol. 2026 May 13;36(10):7572–83. doi: 10.1007/s00330-026-12615-3 (PMC13710651; doi:10.1007/s00330-026-12615-3)
Supplement: Supplementary file 1 [file 330_2026_12615_MOESM1_ESM.pdf]

CT-guided injection in coccydynia: Outcome prediction using MRI findings and CT-injection characteristics

ELECTRONIC SUPPLEMENTARY MATERIAL

Eur Radiol (2026) Kajdi GW; Marth T; Germann C; Farshad M; Sutter R

Supplementary Table S1: Sequence parameters at 1.5T and 3T.

**1.5T MRI**				
Sequence	T1 TSE sag	STIR sag	T1 TSE cor	STIR tra
TR [ms]	599	4020	599	4540
TE [ms]	11	40	11	40
Bandwidth [Hz/px]	181	191	181	191
Slices [n]	25	25	23	29
Slice thickness [mm]	5	5	3	4
Spacing [mm]	5.5	5.5	3.6	4.8
Matrix	384x269	288x230	368x258	288x230
FOV [mm]	200	200	220	200
TA [min:s]	1:06	0:56	1:04	1:26
**3T MRI**				
Sequence	T1 TSE sag	STIR sag	T1 TSE cor	T2 TSE FS tra
TR [ms]	550	5500	550	3500
TE [ms]	9.8	30	9.8	68
Bandwidth [Hz/px]	231	200	231	181
Slices [n]	25	25	18	32
Slice thickness [mm]	5	5	2	4
Spacing [mm]	5.5	5.5	2.4	4.8
Matrix	400x320	320x256	400x320	384x307
FOV [mm]	200	200	220	199
TA [min:s]	1:06	1:44	1:06	1:46

Hz, Hertz; FOV, field of view; FS, fat saturated; sag, sagittal; STIR, short tau inversion recovery; px, pixel; TA, acquisition time; TE, echo time; TR, repetition time; tra, transversal; TSE, turbo-spin-echo.

Supplementary Table S2: Exploratory univariable logistic regression analyses of MRI findings and CT-procedural characteristics

Variable	OR	95% CI	p-value
**Clinical variables**			
Age (per year)	1.00	.96-1.04	.95
Sex (reference female)	.48	.17-1.35	.16
BMI (kg/m^2)	1.02	.93-1.12	.68
Injector experience (per year)	.96	.81-1.13	.60
**Individual imaging findings**			
Modic Type I changes	2.34	.85-6.45	.10
Endplate irregularities and spondylophytes	2.57	.76-8.67	.13
Sacro-/intercoccygeal FSS-hyperintensity	3.57	1.25-10.19	**.02**
Postacchini classification (coccyx morphology) (reference: Postacchini Type I)	1.41	.51-3.9	.50
Sacro-/intercoccygeal subluxation	4.04	1.24-13.23	**.02**
Sacrococcygeal fusion	2.67	.89-7.96	.08
Intercoccygeal fusion	1.2	.39-3.97	.71
Sacrococcygeal angle	1.01	.98-1.05	.53
Intercoccygeal angle	1.00	.98-1.02	.89
Posterior bony spicule	4.59	1.41-14.97	**.01**
Soft-tissue edema and/or bursitis	2.02	.74-5.52	.17
Imaging-negative phenotype	.35	.06-1.94	.23
**Injection target characteristics**			
Synchondrosis vs. apex region	.23	.03-2.2	.21
Direct vs periosteal approach (synchondrosis only)	.90	.31-2.7	.85
**Contrast agent distribution pattern** (reference: posterior contrast distribution)			
Posterolateral	1.13	.38-3.38	.83
Intraligament	.52	.12-2.9	.39

FSS; fluid-sensitive sequence

Supplementary Table S3: Proportion of responders and non-responders (n=63).

Pain relief	Immediate response (15 minutes post-injection)		One-month response (follow-up)		Pain relief ≥ 3 months (follow-up)	
	**n**	**%**	**n**	**%**	**n**	**%**
**Responders**	**35**	**55.6**	**41**	**65.1**	**32**	**50.8**
≥50% to < 75%	10	15.9	12	19.1	N/A	N/A
75% to <100%	12	19.1	10	15.8	N/A	N/A
100%	13	20.6	19	30.2	N/A	N/A
**Non-responders**	**28**	**44.4**	**22**	**34.9**	**31**	**49.2**
> 0% to <50%	19	30.2	3	4.8	N/A	N/A
0%	7	11.1	19	30.1	N/A	N/A
Increased pain	2	3.1	0	0	N/A	N/A
